# Draft Genome Sequence of Haemophilus haemolyticus Strain 16/010 O, Isolated from a Sputum Sample from a Cystic Fibrosis Patient

**DOI:** 10.1128/MRA.00243-19

**Published:** 2019-06-06

**Authors:** Ad C. Fluit, Jumamurat R. Bayjanov, Michael Tunney, J. Stuart Elborn, Malbert R. C. Rogers, Anita C. Schürch, Miquel B. Ekkelenkamp

**Affiliations:** a Department of Medical Microbiology, University Medical Center Utrecht, Utrecht, the Netherlands; b Queen’s University Belfast, Belfast, United Kingdom; Broad Institute of MIT and Harvard University

## Abstract

Haemophilus haemolyticus is considered a commensal of the respiratory tract that can cause opportunistic infections. It is closely related to Haemophilus influenzae. Here, we report the genome sequence of H. haemolyticus 16/010 O, which was isolated from sputum from a cystic fibrosis patient.

## Abstract (precis)

Haemophilus haemolyticus is considered a commensal of the respiratory tract that can cause opportunistic infections. It is closely related to Haemophilus influenzae.

## ANNOUNCEMENT

Haemophilus haemolyticus is a Gram-negative bacterium that is closely related to Haemophilus influenzae (1). It is primarily considered to be a human respiratory tract commensal; it can cause invasive disease and has also been isolated from blood (2). Although (draft) whole-genome sequences for more than 40 strains have been published, a source was available for only 14, and 9 of them were obtained from the sputum or nasopharynx of patients with chronic obstructive pulmonary disease (COPD) (3). Here, we report the draft whole-genome sequence of H. haemolyticus 16/010 O. This strain was cultured from a sputum sample from a cystic fibrosis patient in Belfast, Northern Ireland, United Kingdom, on BCA medium (chocolate blood agar containing bacitracin) at a temperature of 35 to 37°C and 5% CO_2_ for 40 to 48 h. Culture on horse blood agar showed that the strain was hemolytic (4).

Bacteria stored at −80°C were cultured overnight at 37°C on sheep blood agar, and one colony was cultured overnight at 37°C in Luria-Bertani broth. Bacterial DNA was isolated with the QIAcube DNeasy blood and tissue kit using an enzymatic lysis protocol (Qiagen, Germany) after pretreatment with 3 μg/ml lysozyme for 30 min at 37°C. A DNA library was prepared using the Illumina Nextera XT kit and protocol (Illumina, CA) and subsequently sequenced on an Illumina NextSeq platform using the 2 × 150-bp sequencing kit. All reads were trimmed with seqtk trimfq version 1.3 (https://github.com/lh3/seqtk) with an error rate threshold of 0.001, and Nextera transposase sequences were removed with Trim Galore version 0.5.0 (https://www.bioinformatics.babraham.ac.uk/projects/trim_galore/). Contigs were assembled with SPAdes Genome Assembler version 3.11.1, and contigs larger than 500 bp with at least 10× coverage were analyzed further. The read length was 150 bp, and the number of reads was 6,150,070. Assembly resulted in 49 contigs with a total length of 1,843,335 bp, an average coverage of 204×, and a GC content of 38.47%. The reported genome size for H. haemolyticus is approximately 2.0 Mb (2).

The assembled contigs were annotated using Rapid Annotation using Subsystems Technology (RAST) (5). Further analysis for the presence of resistance genes was performed with ResFinder version 3.1 from the Center for Genomic Epidemiology (DTU, Denmark) (6).

Gene annotation using RAST 2.0 identified 1,805 coding sequences and 51 RNAs. With both RAST and ResFinder, no acquired antibiotic resistance genes were found. RAST identified the presence of 8 phage-related proteins. Further analysis showed that 3 contigs harbored contiguous stretches of phage-related genes at the end of contigs of approximately 33, 8, and 7 kb. Genes encoding a putative integrase, a capsid, and tail proteins were identified, suggesting that the strains may carry a bacteriophage. The strain encodes a hemolysin, which may explain the hemolytic phenotype.

### Data availability.

This whole-genome shotgun project has been deposited at DDBJ/ENA/GenBank under the accession number RWKG00000000 and assembly number GCF_004362455. The version described in this paper is the first version, RWKG01000000. The reads can be accessed under SRA accession number SRR8590614 and BioSample number SAMN10405313.
